# Supplementary material for: The evolutionary origin of bilaterian smooth and striated myocytes
Source: eLife. 2016 Dec 1;5:e19607. doi: 10.7554/eLife.19607 (PMC5167519; doi:10.7554/eLife.19607)
Supplement: Supplementary file 1. — (A) Simplified Maximum Likelihood (ML) tree for Myosin Regulatory Light Chain (full tree in panel M), rooted with Calmodulin, which shares an EF-hand calcium-binding domain with MRLC. (B) ML tree for FoxF, rooted with FoxQ1, the probable closest relative of the FoxF family (Shimeld et al., 2010). (C) MrBayes tree for bilaterian ZASP/LBD3, rooted with the cnidarian ortholog (Steinmetz et al., 2012). (D) ML tree for bilaterian Myosin Heavy Chain, rooted at the (pre-bilaterian) duplication between smooth and striated MHC (Steinmetz et al., 2012). (E) MrBayes tree for Mef2, rooted by the first splice isoform of the cnidarian ortholog (Genikhovich and Technau, 2011). (F) MrBayes tree for Titin, rooted at the protostome/deuterostome bifurcation (Titin is a bilaterian novelty). (G) MrBayes tree for Troponin T, rooted at the protostome/deuterostome bifurcation (Troponin T is a bilaterian novelty). (H) MrBayes tree for Troponin I, rooted by the Calponin/Transgelin family, which shares an EF-hand calcium-binding domain with Troponin I. (I) MrBayes tree for MyoD, rooted at the protostome/deuterostome bifurcation (MyoD is a bilaterian novelty). (J) MrBayes tree for Myocardin, rooted at the protostome/deuterostome bifurcation (the Drosophila myocardin ortholog is established [Han et al., 2004]). (K) Complete MRLC tree. Species names abbreviations: Pdu: Platynereis dumerilii; Xenla: Xenopus laevis; Mus: Mus musculus; Hsa: Homo sapiens; Dre: Danio rerio; Gga: Gallus gallus; Dme: Drosophila melanogaster; Cte: Capitella teleta; Patvu: Patella vulgata; Brafl: Branchiostoma floridae; Nve or Nemv: Nematostella vectensis; Acdi: Acropora digitifera; Expal: Exaiptasia pallida; Rat: Rattus norvegicus; Sko: Saccoglossus kowalevskii; Limu or Lpo: Limulus polyphemus; Trib or Trca: Tribolium castaneum; Daph: Daphnia pulex; Prcau: Priapulus caudatus; Cgi or Cgig: Crassostrea gigas; Ling or Linan: Lingula anatina; Hdiv: Haliotis diversicolor; Apcal or Aca: Aplysia californica; Spu: Strongylocent [file elife-19607-supp1.pdf]

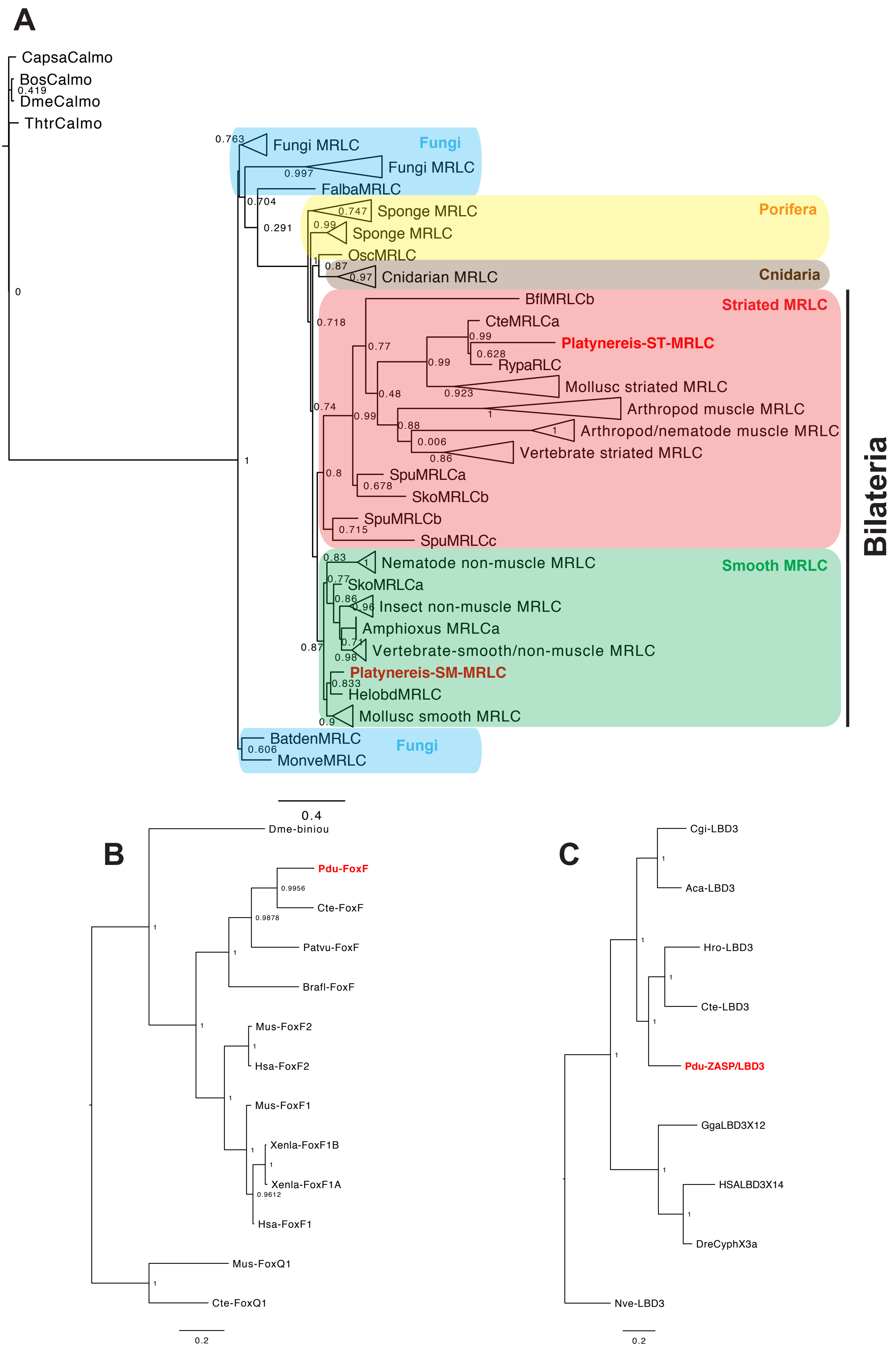

Brunet et al. Supplementary Figure 1

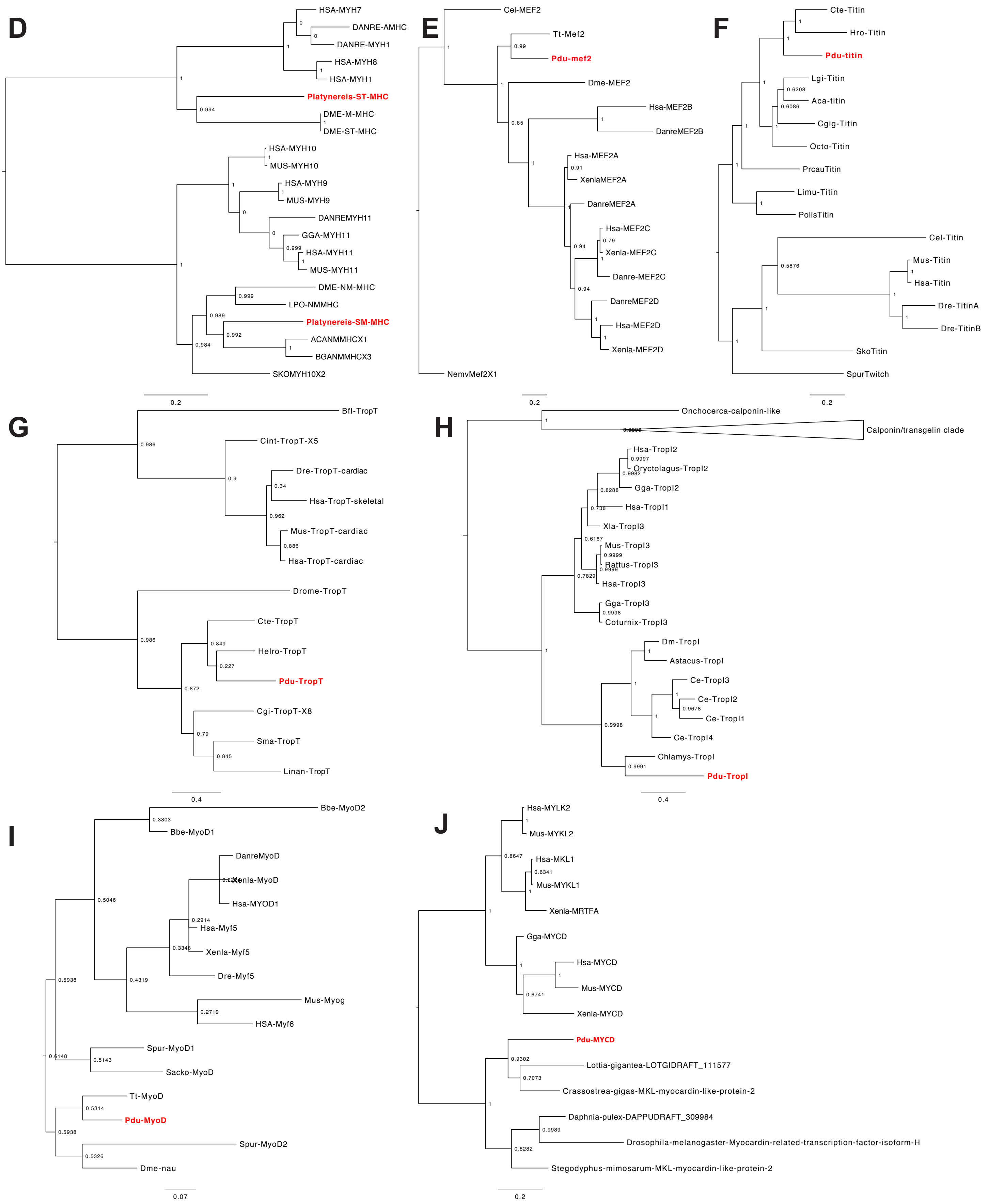

Brunet et al. Supplementary Figure 1

K

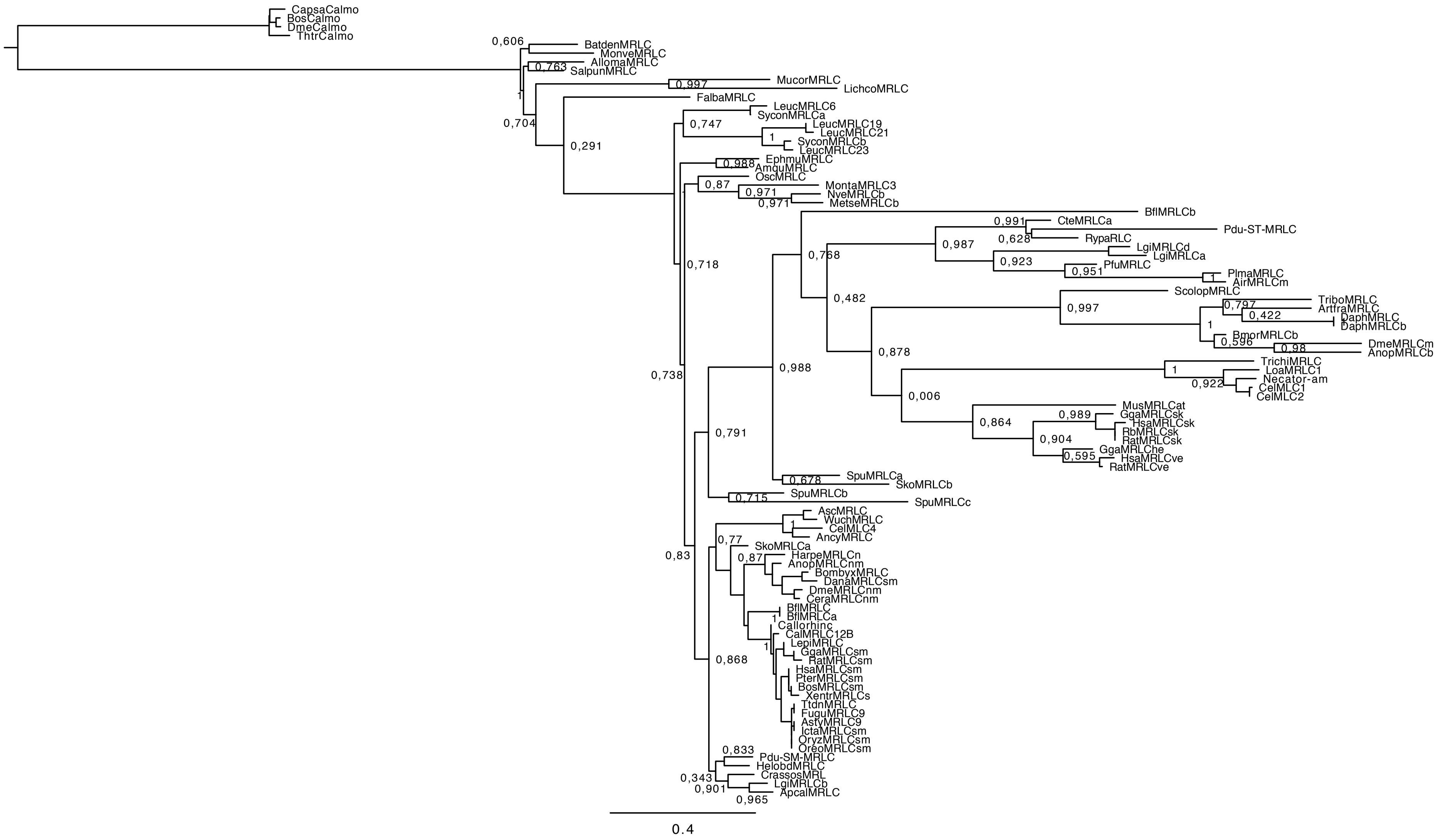

Brunet et al. Supplementary Figure 1
